# Building CRISPR-Based Gene-Editing Platforms for Personalized Medicine: The Next Step in Interventional Genetics

**DOI:** 10.3390/genes17060631

**Published:** 2026-05-30

**Authors:** Sebastian Hernandez Rodriguez, Toshifumi Yokota

**Affiliations:** Department of Medical Genetics, University of Alberta, Edmonton, AB T6G 2H7, Canada

**Keywords:** CRISPR, gene editing, monogenic disorders, interventional genetics, personalized medicine

## Abstract

Recent advances in CRISPR technology have expanded beyond traditional double-strand break–based genome editing to include base editors and prime editors, enabling precise and programmable sequence modifications. This evolution marks a shift from conventional mutation correction toward platform-based therapeutic systems capable of targeting a broad spectrum of pathogenic variants. Such versatility holds promise for addressing a substantial proportion of known disease-causing mutations in rare monogenic disorders. This review discusses the technological progression of CRISPR systems, highlighting the principles, applications, and limitations of emerging editing modalities. We will explore their translation into personalized gene therapies, emphasizing delivery challenges, off-target safety, and the need for regulatory innovation. The paper will also introduce the concept of interventional genetics, an emerging medical framework linking genomic diagnosis directly to therapeutic intervention through adaptive gene-editing platforms. Finally, we will outline strategies for establishing unified, scalable, and regulatory-ready editing platforms that can accelerate the clinical implementation of individualized therapies for rare diseases.

## 1. Introduction

Monogenic disorders arise from pathogenic variants in single genes. Despite their individually low prevalence, these disorders collectively affect approximately 4% of the human population [1]. The low prevalence of individual conditions, combined with their biological complexity and limited commercial incentives, has significantly constrained therapeutic development, leaving many patients without access to appropriate treatments and placing a considerable economic burden on healthcare systems [2,3,4]. In this context, different gene-editing systems, such as transcription activator-like effector nucleases (TALENs), zinc-finger nucleases (ZFNs), and CRISPR-based technologies, have emerged as promising strategies to address the underlying genetic causes of rare monogenic diseases [5,6,7]. Protein-based nuclease technologies such as ZFNs and TALENs have proven to be safe and specific; however, they are more difficult to design and more costly to produce, as their specificity is mediated by protein–DNA interactions, limiting their therapeutic applicability and scalability [7,8,9]. In contrast, CRISPR platforms are inherently modular and reprogrammable, allowing correction of distinct pathogenic variants across diverse disorders and offering durable therapeutic effects from a single intervention [10,11]. Importantly, recent advances in CRISPR engineering, including the development of base editors and prime editors, have enabled more precise and predictable sequence modifications, overcoming several limitations associated with conventional double-strand break–dependent approaches [12]. Moreover, these recently developed CRISPR systems can be engineered to treat both recessive and dominant monogenic disorders, which require distinct gene-editing strategies [13]. In this case, recessive conditions typically require correction of the pathogenic mutation, whereas dominant conditions often require disruption of the gene or its regulatory sequences to silence harmful gain-of-function proteins [13]. Together, these technological advancements have prompted renewed consideration of how personalized CRISPR-based gene-editing therapies could be systematically developed and implemented as standard-of-care interventions for rare diseases, contributing to the emergence of a new clinical therapeutic field referred to as interventional genetics [14]. This review provides a comprehensive overview of the applicability of CRISPR-based gene-editing technologies for the treatment of monogenic diseases. Additionally, we aim to highlight the current status of CRISPR gene-editing therapeutics for rare monogenic diseases, and to preview the roadmap towards a new era of personalized medicine. Finally, we discuss current challenges in the development of CRISPR-based gene-editing therapeutics and outline new strategies to accelerate the clinical implementation of individualized therapies for monogenic diseases.

## 2. CRISPR Mechanisms of Action and Toolkits

Clustered regularly interspaced short palindromic repeats (CRISPR) and CRISPR-associated enzymes (Cas) constitute a naturally occurring adaptive immune system that protects bacterial and archaeal organisms from exogenous nucleic acids [15,16,17,18]. The mechanism of action of the CRISPR-Cas immune response involves three key steps: adaptation, expression, and interference [15,16,19]. In the adaptation phase, short exogenous viral DNA fragments, known as spacers, are captured and integrated into the CRISPR locus [15,16,19]. Next, a single transcript of the CRISPR array carrying the spacers, called pre-CRISPR RNA (pre-crRNA), is transcribed and subsequently undergoes a series of modifications, ultimately maturing into distinct CRISPR RNAs (crRNAs) [15,16,19]. Finally, in the interference phase, the generated crRNA serves as a reference guide for Cas enzymes to recognize the exogenous sequence for cleavage and inactivation [15,16,19].

CRISPR systems can be classified into two main classes according to the organization of the effector modules [17]. Class 1 systems are composed of multiple Cas protein effector modules, while class 2 systems consist of single-multidomain Cas effector modules and occur predominantly in bacteria [15,17,18]. CRISPR-Cas class 2 systems have been widely studied due to their potential in gene-editing and genetic screening applications, with the CRISPR/Cas9 system being the most recognized and widely adopted for genetic engineering [10,20]. As a result, CRISPR-Cas nucleases have been engineered to mediate targeted genetic alterations across diverse animal and cell systems [18]. This versatility has driven the development of CRISPR as a powerful genome editing tool and its implementation as an emerging therapeutic for genetic disorders [10,21]. In this case, the key to this adaptability lies mainly in the ease of programmability of Cas enzymes, which use a single-guide RNA (sgRNA) to direct the Cas protein to a specific genomic target near a PAM (protospacer adjacent motif) region [17,18].

### 2.1. Double-Strand Break-Dependent CRISPR Systems

Traditional gene-editing CRISPR systems, such as CRISPR/Cas9, work by searching for a specific genomic sequence and inducing a double-stranded DNA break (DSB) at the targeted region via the Cas enzyme’s nuclease activity [10]. Following the DSB, the cleaved region can be repaired by non-homologous end-joining (NHEJ) or homology-directed repair (HDR) [10,22]. In the first mechanism, NHEJ can generate small insertions and deletions (indels), enabling highly efficient gene disruption [22,23]. In contrast, the HDR mechanism can result in precise modifications, as a specific DNA sequence is inserted using a homologous DNA template [23,24]. However, inducing efficient HDR remains a critical challenge, as it relies on slower kinetics than NHEJ and is restricted to mitotically active cells (in S and G2 phases), limiting its efficiency in quiescent and postmitotic cells [22]. Several strategies have been developed to improve HDR efficiency, including the addition of short RAD51-binding DNA sequence modules to single-stranded DNA donors [25]. However, another critical limitation of traditional DSB-based CRISPR systems is that DSBs can cause cytotoxicity, p53 pathway activation, and large chromosomal rearrangements [26,27].

### 2.2. Base-Editing Systems

In parallel, other CRISPR-based technologies, such as base editors, offer a new way to induce small and precise genetic modifications without requiring DSBs [28]. Base editing involves inducing single-base transition mutations using base editors to correct or introduce genomic mutations [29]. The most common and widely characterized classes of base editors are adenine base editors (ABEs), which install A-T to G-C base-pair conversions, and cytosine base editors (CBEs), which install C-G to T-A conversions [10,29,30]. Both classes of base editors share a similar structure, where a deactivated Cas enzyme (dCas) or a nickase Cas9 enzyme (nCas), unable to catalyze DSBs, is conjugated to a DNA deaminase enzyme and a sgRNA molecule that provides specificity for the target site [29,30]. nCas enzymes have largely replaced dCas enzymes, given that target strand nicking has been shown to activate cellular repair pathways, thereby improving editing efficiency [31,32]. Base editing depends on the hybridization of the sgRNA spacer to the target sequence, creating an R-loop in which nucleotides on the non-target strand distal to the PAM are exposed as ssDNA [30,33]. This exposure renders them accessible to the base editor’s deaminase domain within a region called the base-editing activity window [30,33]. However, a current challenge is that base editors cannot distinguish targeted from non-targeted bases when multiple editable bases are present within or near this activity window, leading to undesired base modifications, referred to as bystander single-nucleotide conversions [34]. Because precise base editing depends on delimiting the activity window, controlled by the base editor’s structure, research has focused on developing a new generation of base editors with narrower activity windows to address bystander single-nucleotide conversions [35]. For example, Tan et al. developed more precise base editors by fine-tuning linker sequences between the deaminase and Cas domains [36].

### 2.3. Prime-Editing Systems

Prime editors are another high-precision genome-editing CRISPR tool that enables all 12 possible nucleotide conversions and small insertions and deletions without requiring DSBs [28,37]. As with base editors, the prime-editing system is built around an nCas enzyme; however, in this context, the nickase is fused to a murine leukemia virus reverse transcriptase (MMLV-RT) and paired with a modified sgRNA designated as a prime-editing gRNA (pegRNA), which serves a dual function by conferring target specificity and encoding the intended genomic modification [10,28,37,38]. Unlike classic sgRNA molecules, pegRNAs are distinguished by an extended 3′ end that harbors both a primer binding site (PBS), complementary to the protospacer sequence, and an RT template that encodes for the desired modification [10,28,38]. Prime editor systems work by introducing a single-strand break in the non-targeted strand via an nCas enzyme, leaving a 3′-ssDNA flap free to hybridize with the PBS of the pegRNA [10,28,37,38]. Hybridization of pegRNA with a 3′-ssDNA flap allows RT to extend 3′-ssDNA using RT template encoded in the pegRNA, producing an edited 3′-ssDNA flap [10,28,37,38]. The extended 3′ ssDNA flap is subsequently incorporated into a heteroduplex, which is resolved through ligation and DNA mismatch repair (MMR), yielding the desired genomic modification [10,28,38]. A major advantage of prime-editing is its reduced off-target activity compared with base editors and DSB-dependent CRISPR systems [27,28,39]. This specificity arises from additional hybridization steps, which further limit unintended editing at off-target sites [28]. However, as with other Cas-derived gene-editing systems, editing efficiency remains a primary limiting factor in prime editing [40]. Consequently, novel strategies have aimed to optimize enzyme domains and pegRNA architecture, such as the incorporation of structured RNA motifs at the 3′ end of pegRNAs, which has been shown to enhance prime editing efficiency across multiple human cell lines [41].

### 2.4. CRISPR Transcriptional Modulator Systems

In addition to base editors and prime editors, other CRISPR/Cas systems have been developed to artificially modulate gene expression [42]. These CRISPR/Cas transcriptional modulation systems fall into two main categories: (1) CRISPR activation (CRISPRa), which mediates transient transcriptional or long-term epigenetic activation, and (2) CRISPR interference (CRISPRi), which mediates transient transcriptional or long-term epigenetic inhibition [43]. The simplest CRISPRi system consists of dCas9, which sterically blocks RNA polymerase from transcribing the targeted gene [43]. However, different transcriptional repressors or activation domains have been linked to dCas9, such as KRAB domains or VP64, expanding the toolbox of CRISPRi and CRISPRa systems [43,44]. For example, CRISPRoff represents a newly developed programmable epigenetic modifier that can induce gene silencing in a stable and heritable manner by establishing DNA methylation and repressive histone modifications [45]. Similar to CRISPR-based gene-editing systems, CRISPRi and CRISPRa platforms hold therapeutic promise because, unlike other CRISPR systems, they can modulate gene expression without altering the patient’s DNA [43,46]. In this review, however, we will focus on the applicability and current progress of CRISPR-based gene-editing systems.

### 2.5. Delivery Systems for CRISPR Machinery

Therapeutic translation of gene-editing systems, such as base and prime editors, requires the efficient delivery of editing molecules (Cas enzymes and sgRNAs/pegRNAs) across both ex vivo contexts (where transplantable cells are targeted) and in vivo settings involving tissues and organs [47,48]. Delivery systems can be classified into two main types: (1) viral vectors and (2) non-viral vectors [47,48]. Viral vectors such as retroviruses, adenoviruses (AdVs), or adeno-associated viruses (AAVs) exploit viral mechanisms to deliver nucleic acids into the cell nucleus, substituting viral genomes with gene-editing molecules [49]. Notably, high-capacity recombinant AdVs (HC-rAdVs) have demonstrated the ability to transduce complete base-editing and prime-editing complexes [47]. More recently, virus-like particles (VLPs) have emerged as an alternative delivery strategy, in which the expression of structural proteins derived from viral capsids or envelopes drives the self-assembly of particles that resemble the outer or whole viral structures, which can be harnessed as delivery vectors [47,50]. Meanwhile, non-viral vectors include electroporation (mainly used for ex vivo therapies), lipid nanoparticles (LNPs), synthetic or polymeric nanoparticles, extracellular vesicles (EVs), and inorganic nanoparticles [47]. In particular, LNPs have been used for various purposes, from mRNA vaccines against SARS-CoV-2 to gene-editing therapies [51]. As a result, LNPs have been actively investigated to design and fine-tune LNPs formulations that can improve encapsulation, stability, and delivery efficiency [47,51].

## 3. Personalized Medicine Applications

**Figure 1:**
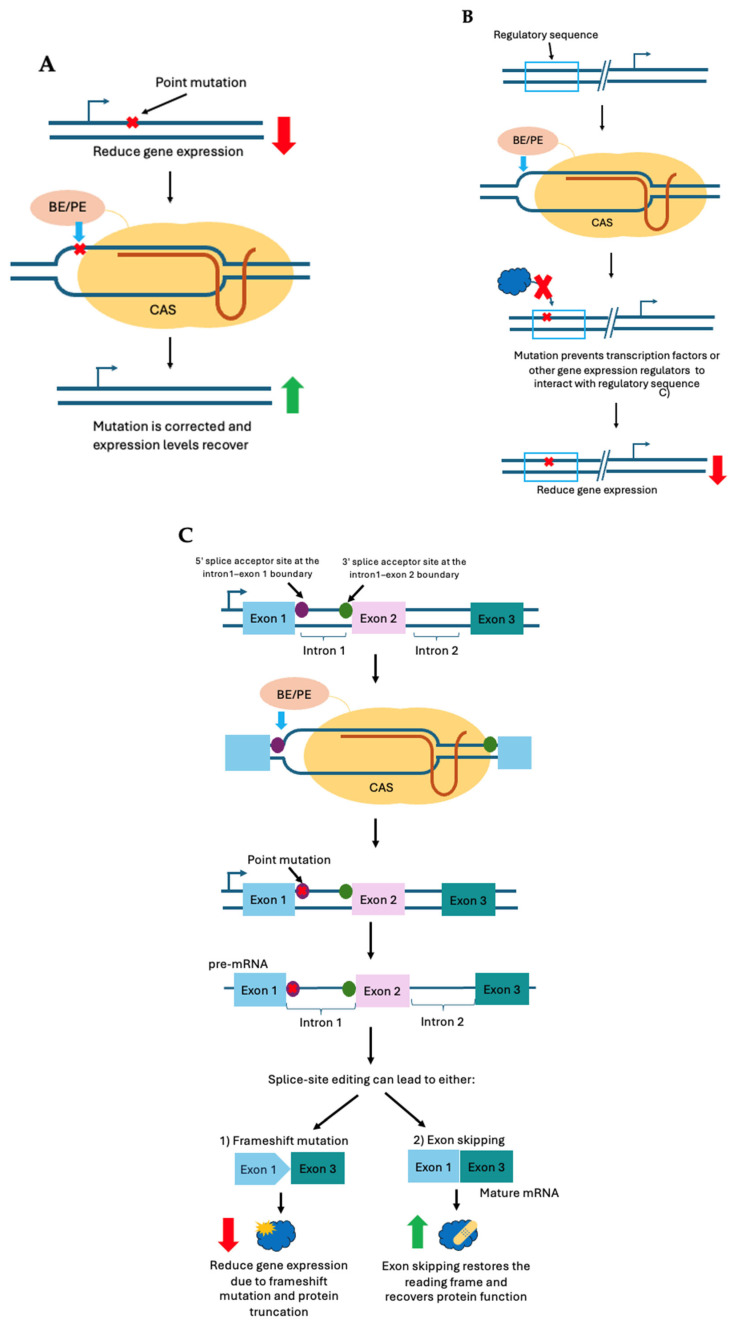
Schematic of different CRISPR gene-editing approaches for the treatment of monogenic diseases. (**A**) Correction of a pathogenic mutation within the gene of interest. The sgRNA guides a Cas enzyme linked to a base editor (BE) or prime editor (PE) to the mutated locus. Upon sgRNA hybridization, BE or PE activity introduces precise nucleotide modifications that restore the normal gene sequence and promote normal gene expression. This approach is primarily used for recessive monogenic disorders, aiming to restore physiological gene expression. (**B**) Targeted mutagenesis of regulatory elements of the gene of interest to modulate its expression. In this approach, the sgRNA targets a regulatory sequence of the gene; following hybridization, BE or PE activity introduces mutations that reduce the expression of the target gene. This strategy is generally used to treat dominant monogenic diseases. (**C**) Targeted editing of splice donor or acceptor sites to alter splicing outcomes. The sgRNA directs a BE or PE to splice regulatory elements, such as the 3′ splice acceptor (green circle) or 5′ splice donor site (purple circle). Editing of these sites can induce exon skipping to restore the reading frame and recover protein function, or disrupt splicing to reduce expression of a pathogenic transcript. Light blue arrows indicate the BE/PE editing sites, green arrows indicate upregulation, and red arrows indicate downregulation of gene expression.

The development of therapeutics to treat rare diseases, referred to as orphan drugs, is a major healthcare challenge, as it is estimated that over 7000 rare diseases encompassing metabolic, neuromuscular, blood, and immunological disorders still lack appropriate treatment [52,53]. In this context, gene-editing therapies based on CRISPR/Cas systems represent a promising strategy for developing new therapeutics for rare monogenic disorders by targeting the root molecular cause of disease [26,52]. Here, non-DSB mediating CRISPR/CAS gene-editing systems, including base editors and prime editors, can induce precise modifications tailored to the desired therapeutic effect and the specific patient mutation [47]. CRISPR-based gene-editing therapies support multiple editing strategies, each tailored to the mutational context of the monogenic disorder being treated (Figure 1). As a result, CRISPR-based gene-editing therapies can correct specific point mutations or small genomic variants to restore normal gene function (Figure 1A) [54]. This is exemplified by the recently reported N-of-1 in vivo base-editing treatment for carbamoyl-phosphate synthetase 1 (CPS1) deficiency [55]. In addition, regulatory elements, such as promoters, can be targeted to modulate expression of either the mutated gene itself or other genes capable of alleviating the disease phenotype (Figure 1B) [56]. For example, an ex vivo cell therapy for sickle cell disease (SCD) modifies the promoters of the HBG1/2 genes to disrupt the BCL11A transcriptional repressor binding sites, leading to increased fetal hemoglobin production [57,58]. Lastly, another editing strategy relies on modifying splicing regulatory elements to induce exon skipping or inclusion, resulting in either restoration of gene function (fully or partially) or complete knockout (Figure 1C). An example is a recent in vivo CRISPR/ABE base-editing therapy for familial hypercholesterolemia, which introduces point mutations at a PCSK9 splice site to disrupt PCSK9 expression [59].

**Figure 2:**
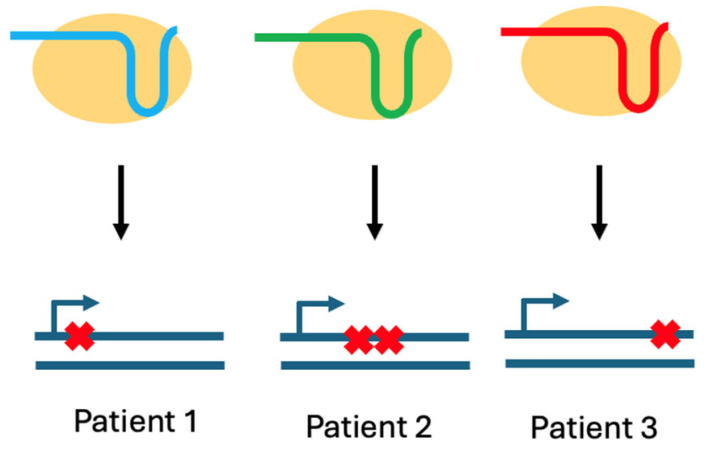
Representation of the versatility and modularity of CRISPR-based gene-editing systems for treating the diverse mutational spectra of monogenic disorders through the design and testing of different sgRNAs. Personalized sgRNAs, together with base editors or prime editors, can be designed according to a specific patient mutation.

Taken together, the intrinsic versatility and programmability of CRISPR/Cas systems hold the potential to tailor therapeutic interventions to each patient’s unique mutational profile, opening the window to a new era of personalized medicine (Figure 2) [58,60]. Therefore, CRISPR/Cas-based platforms represent a compelling strategy for developing personalized, single-dose, and long-lasting treatments for rare monogenic disorders [20,54]. Beyond monogenic conditions, CRISPR/Cas gene-editing therapies also show potential for addressing polygenic human diseases, including cancer, through approaches such as CAR-T-cell immunotherapy, as well as for neurological disorders such as Alzheimer’s and Parkinson’s disease [26,61].

## 4. CRISPR-Based Gene-Editing Therapy Case Studies

Over recent years, genomic medicine has accelerated the investigation of novel genetic therapies to treat rare diseases [62]. Further progress has been driven by the implementation of incentive policies that fund research and support N-of-1 (single-subject) clinical trials for ultra-rare monogenic diseases, such as the Food and Drug Administration (FDA) orphan drug designation policy, collectively driving the development of new genetic therapeutic strategies [63,64]. Here, as previously discussed, the greatest potential of CRISPR-based gene-editing treatments lies in their versatility to induce precise modifications to address different monogenic diseases and the patients’ mutational heterogeneity within specific diseases. This therapeutic potential is now being evaluated in several clinical and early-access settings, including the examples summarized below. Table 1 summarizes selected CRISPR-based gene-editing therapies currently being evaluated in clinical trials or early-access settings.

### 4.1. Base Editing for the Treatment of CPS1 Deficiency

Carbamoyl-phosphate synthetase 1 (CPS1; MIM # 237300) is a critical and rate-limiting enzyme in the urea cycle, which catalyzes the condensation of ammonia and bicarbonate into carbamoyl-phosphate [65]. CPS1 deficiency is an autosomal recessive monogenic urea cycle defect (UCD) caused by mutations in the *CPS1* gene located at 2q34 [65,66]. CPS1 deficiency is extremely rare, with an estimated prevalence of 1 in 1,300,000 people [66]. Moreover, CPS1 deficiency can be classified by symptom onset as neonatal or late-onset [67]. Neonatal CPS1 deficiency is characterized by more severe symptoms that can be life-threatening [67]. Recently, a personalized lipid nanoparticle-delivered CRISPR base-editing therapy, kayjayguran abengcemeran, was used to correct the Q335X variant (c.1003C→T) in the *CPS1* gene present in a neonatal patient [55]. Here, in a single-patient, expanded-access investigational new drug application, an adenine base editor (NGC-ABE8eV106W) mRNA and a gRNA targeting the adenine in the eighth position of its protospacer sequence to rescue the Q335X variant were encapsulated in lipid nanoparticles and then administered to the patient in two doses [55]. Early findings showed that treatment with this personalized base-editing therapy enabled the patient to increase his protein intake and reduce the nitrogen scavenger medication used to lower ammonia levels, suggesting patient improvement [55]. However, further studies are yet to determine the safety profile and long-term effects of the therapy, as well as whether germline editing occurred in the patient.

### 4.2. Base Editing for the Treatment of Duchenne Muscular Dystrophy

Duchenne muscular dystrophy (DMD; MIM # 310200) is a recessive X-linked genetic disorder characterized by progressive muscle degeneration [68]. DMD is caused by mutations in the *DMD* gene located on chromosome Xp21, which encodes dystrophin, a critical protein for muscle cells [68]. DMD-causing mutations are primarily nonsense and frameshift mutations that disrupt the reading frame of *DMD* transcripts, resulting in the absence of functional dystrophin in muscle cells due to protein truncation [68]. Importantly, approximately 25–35% of patients carry point mutations that could be rescued by patient-tailored base-editing therapies [69]. Beyond correcting point mutations, base-editing approaches can also modulate splicing by targeting splicing regulatory elements, thereby promoting the skipping of specific exons to restore the reading frame and recover dystrophin function in the mutant *DMD* gene, a strategy analogous to that employed by currently approved antisense oligonucleotide therapies for DMD [69,70].

HG302 is a base-editing therapy under a Phase 1 clinical trial (NCT06594094) developed by HuidaGene Therapeutics [71]. HG302 is a single-AAV-delivered CRISPR/hfCas12Max system designed to skip exon 51 by targeting the exon 51 splice donor site [72]. Notably, this therapy holds great potential, as it is estimated that ~14% of DMD patients can be treated by skipping exon 51 [73]. HG302 preclinical testing in humanized DMD mice showed significant dystrophin restoration in muscle fibers and improved muscle function [71]. Although the study is completed, the results of the Phase 1 clinical trial have yet to be formally reported.

Another therapeutic recently developed is GEN6050X, which is a cytosine-based editing therapeutic delivered by a dual single-stranded adeno-associated virus serotype 9 (ss.AAV9) vector [74]. GEN6050X targets the skipping of exon 50 by modifying the 5′ splicing site of exon 50 to rescue the reading frame, which is lost due to the complete deletion of exon 51, multiexon deletions, or small mutations within exon 50. Findings of the Phase 1 clinical trial (NCT06392724) have yet to be posted [74]. However, preclinical studies using induced pluripotent stem cell-derived cardiomyocytes carrying an exon 51 deletion showed that base editing induced exon 50 skipping in a high proportion of DMD transcripts and restored dystrophin production with functional properties similar to full-length dystrophin [75].

### 4.3. Prime Editing for the Treatment of p47phox-Deficient Chronic Granulomatous Disease

Chronic granulomatous disease (CGD; MIM # 306400, 233700, 233710, 613960, 233690, & 618935) is a rare monogenic immunodeficiency disease affecting approximately 1 in 200,000 people in the US [76]. CGD is caused by mutations in genes that are critical for proper NADPH oxidase activity (*CYBB, CYBA, NCF1, NCF2, NCF4, RAC1, RAC2, or CYBC1*) [77]. NADPH oxidase is a multimeric enzyme complex composed of several protein subunits (gp91phox, p22phox, p40phox, p47phox, p67phox, and GTPase RAC), that plays a critical role in producing superoxide anion to kill microorganisms in phagocytic leukocytes [76,77]. As a result, patients with CGD have a high susceptibility to acquiring infections, which can be life-threatening. Autosomal recessive p47phox-deficient CGD is caused by mutations in the *NCF1* gene encoding p47phox, a cytosolic component of NADPH oxidase [76]. CGD caused by p47phox deficiency accounts for 25% of CGD patients, where a two-nucleotide deletion in exon 2 (ex2delGT) is the most common mutation found in ~80% p47phox-deficient CGD patients [78]. Recently, an ex vivo stem cell therapy called PM359 was developed in which autologous CD34+ hematopoietic stem cells are modified via prime-editing to correct ex2delGT in the *NCF1* gene or its pseudogenes (NCF1B and NCF1C) or both [78]. This represents the first prime editing therapy tested in humans, where an open-label, single-arm, multicenter Phase 1/2 study (NCT06559176) is currently active to assess its safety and efficacy in p47phox CGD patients [79]. The latest shared results showed that treatment with autologous prime-edited CD34+ cells in two patients restored NADPH oxidase activity and corrected neutrophil function [78]. Further observation of treated patients is needed to confirm sustained therapeutic effects and the therapy’s safety profile.

### 4.4. Base-Editing for the Treatment of Familial Hypercholesterolemia

Familial hypercholesterolemia (FH; MIM #143890, 144010, 603813, & 603776) is an autosomal dominant disorder characterized by elevated low-density lipoprotein cholesterol (LDL-C) levels and a high risk of developing atherosclerotic cardiovascular disease (ASCVD) [80,81]. FH is the most prevalent genetic disorder of lipid metabolism affecting around 1 in 250–300 individuals. FH can be classified by the number of pathogenic variants into heterozygous FH (single pathogenic variant and late disease onset) and homozygous FH (two pathogenic variants and early disease onset) [80,81]. Heterozygous FH is commonly caused by mutations in three genes involved in the LDL-C clearance pathway: *LDLR* (the most common cause in patients), *APOB*, and *PCSK9* [82]. PCSK9 is a key regulator of cholesterol metabolism that binds to the LDL receptor and promotes its degradation, ultimately leading to elevated plasma LDL-C levels [83].

VERVE-101 and its successor VERVE-102 represent newly developed CRISPR-based base-editing therapeutic candidates aiming to inactivate *PCSK9* expression in the liver by introducing a precise modification at the *PCSK9* splice donor site, with the ultimate goal of reducing plasma LDL-C levels [84,85]. VERVE-102 consists of an adenine base editor mRNA paired with a guide RNA targeting the *PCSK9* locus and is delivered via a GalNAc-conjugated lipid nanoparticle (LNP) designed for liver-specificity, mediating an A-T-to-G-C nucleotide conversion at the PCSK9 splice donor site [84,85]. Here, a Phase I clinical trial (NCT05398029) administered VERVE-101 (the predecessor of VERVE-102, not conjugated with GalNAc) at different dosages to heterozygous FH patients, showing significant reductions in both PCSK9 and LDL-cholesterol levels [86,87]. However, in April 2024, Verve Therapeutics (sponsor) announced that it would halt enrollment in this clinical trial after a patient who received 0.45 mg/kg of VERVE-101 experienced a serious drug-related adverse event (ALT elevation and thrombocytopenia) [86,87]. Currently, a Phase 1 clinical trial (NCT06164730) is evaluating the safety and efficacy of VERVE-102. Interim data from the company showed an LDL-C dose-dependent reduction, with an average 53% reduction in the 0.6 mg/kg dose group, and no serious drug-related adverse events have been reported [88].

Moreover, another base-editing therapy, YOLT-101, induces an A-to-G substitution that disrupts *PCSK9* mRNA splicing and silences the *PCSK9* gene [89]. Similar to VERVE-102, YOLT-101 consists of a PCSK9-targeting gRNA and mRNA encoding hpABE5, encapsulated in GalNAc-LNPs for specific liver delivery [89]. Here, a Phase 1 clinical trial (NCT06458010) is underway to evaluate the safety and tolerability of YOLT-101 in patients with heterozygous FH [89]. An early report of the clinical trial shows that a single treatment with YOLT-101 induced dose-dependent, long-lasting reductions in circulating PCSK9 and LDL-C of 74.4% and 52.3%, respectively, in the 0.6 mg/kg cohort [89]. However, infusion-related reactions and elevations in liver enzymes were reported as drug-related adverse events, suggesting that close monitoring of patients is needed to ensure safety.

## 5. Challenges in Platform Development of CRISPR-Based Gene-Editing Therapies

Despite the great potential and significant progress in CRISPR-based gene-editing therapies for the treatment of monogenic disorders, four critical challenges remain: (1) delivery barriers, (2) off-target effects, (3) ethical concerns, and (4) scalability and regulatory legislation.

### 5.1. Delivery Barriers

CRISPR-based gene-editing therapies rely on the delivery of genetic material (sgRNAs and mRNAs encoding the editing machinery) into target cells. However, a major challenge with these therapeutics is effective intracellular delivery, since nucleic acids are susceptible to degradation and cannot cross the lipid bilayers of the cell membrane or the subcellular compartments, such as the nucleus [11,90]. Among non-viral delivery systems, electroporation stands out for its high transfection efficiency, making it the method of choice for ex vivo therapies [11]. For in vivo applications, however, it is not viable, and alternative delivery systems (viral and non-viral) must be employed, each carrying its own set of advantages and limitations [91].

#### 5.1.1. Viral Vectors

Viral vectors, such as rAAVs and lentiviruses, have received regulatory approvals for the treatment of various monogenic disorders, including DMD and hemophilia B, due to their relatively low immunogenicity across patients [48,92]. However, a major challenge lies in the fact that viral vector-delivered therapies may have a ‘one-try’ limitation because immune responses after initial exposure can limit redosing, and pre-existing immunity may also restrict eligibility for some patients [93]. Moreover, another major limitation concerns long-term efficacy and safety, as viral vectors can still induce immunogenic responses and persist in cells via double-stranded circular episomes that can be lost after several mitotic events [94]. In this context, new modifications to different rAAV vectors have been studied to optimize effect longevity and safety, such as reducing CpG levels in the vector genome, since it has been reported that CpG dinucleotides within the transgene can induce innate Toll-like Receptor 9 (TLR9)-mediated cytotoxic CD8+ T-cell response [95,96]. Another major concern with viral vectors is the risk of insertional mutagenesis, which can drive the formation of malignancies [97]. This is particularly relevant for viruses that integrate their DNA into the host genome during their life cycle, such as lentiviruses [97]. Here, several modifications to the viral genome have been implemented to reduce the risk of insertions, such as deletions in the 3′ long terminal repeat (LTR) in lentivirus vectors to produce self-inactivating lentiviral vectors [98]. However, a limited number of malignancies following viral vector-mediated delivery have been reported, some of which have been attributed to external factors [48,98]. Nevertheless, continued monitoring of patients treated with viral vector-based therapies is needed to comprehensively assess their long-term safety profile.

#### 5.1.2. Lipid Nanoparticles

LNPs represent another major vehicle system for delivering nucleic acids into cells [90]. LNPs have been widely used in recent mRNA vaccines and gene-editing therapies due to their low immunogenicity, protection against degradation, targeted delivery via conjugation with targeting ligands, and simple preparation [51,90,99]. However, because LNPs enter the cell via endocytosis, endosomal escape of LNP contents remains a central barrier to efficient nucleic acid delivery, as it is estimated that only 1–2% of internalized nucleic acids reach the cytoplasm [100]. In this context, research must focus on designing more efficient formulations that yield higher endosomal release rates [11,90,101]. For example, Li et al. investigated a new LNP design using ionizable lipid-coated gold nanoparticles (IC-AuNPs), which led to more efficient endosomal escape and cytoplasmic mRNA diffusion [102]. Another critical challenge with LNPs is that some of their components, such as polyethylene glycol (PEG) lipids, have been reported to induce immunogenic and anaphylactic reactions [103]. Thus, further immunological studies elucidating the complete mechanisms underlying LNP-induced immune responses are needed to develop new strategies and LNP designs that can modulate the immune response against LNPs. Finally, LNPs have an intrinsic liver-specificity due to their association with certain serum proteins, like apolipoprotein E, that direct them to receptors in liver cells [101]. As a result, LNPs accumulate mainly in the liver, which can reduce therapeutic efficacy and induce hepatotoxicity driven by liver sinusoidal endothelial cells and Browicz–Kupffer cells [104]. Here, new LNPs formulations, such as zwitterionic pyridine carboxybetaine ionizable lipids, have been reported to reduce liver accumulation by ~70% in animal models [105]. Suggesting that the investigation of new LNPs formulations can address this critical limitation.

### 5.2. Off-Target Effects

A major concern in the clinical translation of CRISPR-based gene-editing therapies is the potential for off-target genotoxicity [106]. Despite sgRNAs providing genomic specificity to the different CRISPR/Cas systems, unintended modifications can occur at other loci due to high sequence identity to on-target sites or tolerance of DNA and RNA base bulges in CRISPR-Cas complexes [106]. This can lead to diverse deleterious genetic modifications, reducing the safety of CRISPR-based gene-editing therapies. Prime editing represents a particularly promising approach in this regard, exhibiting minimal off-target effects relative to other CRISPR-based platforms [31,32]. This is supported by different studies, which have found that prime-editing systems induce minimal off-target effects [39,107]. Moreover, this improved specificity in prime editors is likely due to the reverse transcriptase domain, which, unlike base editors, does not produce gRNA-independent off-target activity, and to the additional hybridization steps required for prime editing, reducing gRNA-dependent mutagenesis [108]. Additionally, novel prime editor-based assays such as TAPE-seq and PEAC-seq have been developed to identify CRISPR off-target candidates, showing high predictive power for genome-wide off-target effects with high validation and low miss rates [109,110]. Beyond prime editing, in silico prediction tools have been developed to design highly specific gRNAs across CRISPR systems, as demonstrated by Zhang et al., who applied deep learning models to predict editing efficiency and potential off-target sites of base editors [111]. In addition, other strategies, such as hybrid gRNAs, which add DNA nucleotides to the spacer sequence, have been shown to be effective at reducing off-target effects [112]. Lastly, the generation of new, more specific and efficient base and prime editors over the past few years has yielded substantial progress in minimizing off-target effects [32].

### 5.3. Ethical Concerns

Another major challenge is the ethical concerns about the accessibility and affordability of these new therapeutics. Recently approved CRISPR-based and gene-targeted therapies can cost several million USD, creating major barriers to access [113,114]. Moreover, therapy access is mainly restricted to high-income and developed countries, resulting in patients living outside these countries without the means to access appropriate treatment [113]. As a result, government and institutional intervention with funds and policies that mitigate the economic burden on patients and/or their families, and support the development and cap the costs of genetic therapies, is required [113,115,116,117]. In this regard, publicly funded institutions like universities should advocate for promoting lower prices in their licensing agreements rather than seeking to maximize returns, as discussed by Joffe et al. [118]. Additionally, new business models that make the costs of CRISPR therapeutics more manageable for payers are needed to ensure equitable access to gene-editing therapies [119,120]. For example, implementing different payment models, such as amortization and risk spreading, as proposed by Horrow and Kesselheim, can help reduce the burdensome upfront costs of gene-editing therapies [119]. Lastly, innovations in manufacturing processes that can reduce the cost of CRISPR-based therapies can also help address this challenge [114]. Here, the introduction of LNPs as a delivery system for gene-editing therapies represents a more cost-effective approach than viral vectors, which, a study showed, accounted for 48% of the cost of currently available gene therapies [121].

### 5.4. Scalability and Regulatory Frameworks

Mutational heterogeneity within monogenic disorders poses a significant scalability challenge for CRISPR-based gene-editing therapies, as each variant requires the development and testing of specific sgRNAs or pegRNAs in dedicated in vitro and/or in vivo models. Therefore, developing new CRISPR-based gene-editing therapies is often both resource-intensive and time-consuming [122]. One promising solution is the generation of multi-variant cell lines capable of evaluating multiple sgRNAs and editing strategies within a single system [122]. For example, Quigley et al. recently reported the development of immortalized HuH-7 human hepatoma cells harboring the six most common phenylketonuria (PKU)-causing variants, enabling streamlined assessment of base editors and sgRNAs across multiple PKU variants simultaneously [122,123].

In addition, the manufacturing process for CRISPR therapeutics represents another major challenge for the clinical application and scalability of gene-editing therapies. Currently, the FDA and other national agencies, such as the European Medicines Agency (EMA), have a set of general guidelines for the development of new human gene therapy and genome editing therapeutics, including information on product design, manufacturing and testing, nonclinical safety assessment, and clinical trial design [124,125]. Here, investigational new drug (IND) applications must provide substantial information and ensure the drug product’s manufacturing quality [124,125]. This creates a major challenge for the production and distribution of CRISPR therapeutics, as good manufacturing practice (cGMP) facilities are scarce and new cGMP facilities require significant investment [114,126].

Furthermore, the establishment of new regulatory policies specifically designed for personalized gene-editing therapies targeting monogenic disorders holds considerable potential to accelerate and reduce the costs associated with the approval process [14,126]. Such policies should provide greater flexibility in conducting clinical trials and enable the integration of a single therapeutic platform that can address multiple patient-specific mutations within a unified application, leveraging the inherent modularity of CRISPR-based tools [14,126]. In this context, in February 2026, the FDA announced a novel regulatory framework for the development of individualized therapies for genetic disorders, encompassing gene-editing therapeutics [127]. This framework permits the initiation of first-in-human (FIH) studies based on sufficient proof-of-concept evidence derived from preclinical investigations, while also allowing for the inclusion of new targets within a single biologics license application (BLA) [127]. Collectively, regulatory initiatives of this nature have the potential to accelerate the development of novel therapeutics and expand access to tailored treatments for patients affected by monogenic disorders for which no cure is currently available.

## 6. Interventional Genetics

The introduction of genetic therapies to treat monogenic disorders revolutionized the clinical field, offering new treatments to patients without options [128]. However, developing therapeutics to address the heterogeneity of mutations and the diversity of monogenic disorders remains a major challenge for meeting treatment demand [129]. Here, CRISPR-based gene-editing therapies represent a promising solution to address this challenge due to their specificity and programmability. As a result, CRISPR-based gene-editing therapies have the potential to serve as a platform for personalized therapeutics to treat monogenic disorders [54,128]. This possibility of generating efficient and safe therapeutics using CRISPR tools and other genetic strategies, such as antisense oligonucleotides, has led to the creation of a new clinical framework called “interventional genetics” [14,130,131]. This medical framework aims to integrate genomic diagnosis with the development and implementation of precise, personalized therapies, including gene-editing therapeutics, based on a patient’s genetic diagnosis [14,130,131]. It is proposed that a multidisciplinary team composed of clinical laboratories, clinicians, researchers, bioethicists, and other specialized members will be capable of diagnosing diseases with a clear genetic cause, interpreting sequencing findings to develop novel therapies for patients without available treatments, and guiding and managing the treatment of patients with newly developed genetic therapies [14,130,131]. In this context, Giannikopoulos et al. proposed establishing an “interventional genomics board” to oversee the entire pipeline, from the evaluation and interpretation of sequencing results through the treatment of patients with next-generation genetic therapies [130]. However, interventional genetics is a relatively new medical framework, and a clear plan to connect all stakeholders involved in the research, regulatory agencies, and the clinical stage is needed to make genetic therapies the new standard of care for monogenic disorders.

## 7. Conclusions

Overall, CRISPR-based gene-editing therapies currently under investigation highlight the versatility of CRISPR platforms for addressing disease-causing mutations across a wide range of monogenic disorders. These therapies represent a potentially transformative approach for conditions that lack effective treatment options. Interventional genetics provides an emerging framework in which molecular diagnosis can be linked directly to therapeutic development and implementation, including CRISPR-based gene editing and other modular platforms such as antisense oligonucleotides. However, successful implementation will require continued optimization of editing tools and delivery systems, long-term safety monitoring, scalable manufacturing, appropriate regulatory pathways, and equitable patient access.

## Figures and Tables

**Table 1:** Overview of CRISPR-based gene-editing therapies currently evaluated through clinical trials and used to treat monogenic diseases. Data were obtained from ClinicalTrials.gov and relevant publications.

Trial ID	Phase	Study Design	Number of Participants	Status	Target Disease and Gene (Mutation, If Applicable)	Delivery System	Brief Description
NCT06860672	Phase 1	Open-label, single-group assignment	1	Recruiting	Snijders Blok–Campeau syndrome Heterozygous mutation (c.3073C>T; p.R1025W) in the *CHD3* gene	Dual Vector AAV	In vivo dual-AAV delivery base-editing therapy. Designed to correct the mutant A-T base pair using a TadA-embedded adenine base editor (TeABE).
NCT06559176	Phase 1/2	Open-label, single-arm, multicenter	12	Recruiting by invitation	Autosomal Recessive Chronic Granulomatous Disease Deletion mutation (c.75_76delGT) in the *NCF1* gene	Electroporation	Prime editing of autologous CD34+ stem cells for ex vivo therapy. Designed to correct the exon 2 del GT mutation in the *NCF1* gene, encoding p47phox protein.
NCT06959771	Phase 1/2	Open-label, single-group assignment	1	Recruiting	X-linked hyper-IgM (HIGM) syndrome Point mutation (c.658C>T; p.Q220X) in *CD40L* gene	Electroporation	Base editing of autologous hematopoietic stem/progenitor cells (HSPC) and T cells (BE T) as ex vivo therapy. Designed to rescue CD40L expression by correcting the c.658C>T point mutation
NCT07176923 & NCT07371767	Phase 1	Open-label, single-arm, dose-escalation	15	Recruiting	Familial chylomicronemia syndrome (FCS) & Hyperchylomicronemia *APOC3* gene.	Lipid nanoparticles	In vivo base-editing therapy delivered by lipid nanoparticles targeting the *APOC3* gene. Designed to introduce mutations that reduce APOC3 expression, lower serum triglyceride levels, and reduce pancreatitis risk.
NCT06325709	Phase 1/2	Open-label, single-group assignment	10	Recruiting	Chronic Granulomatous Disease Missense mutation (c.676C>T) in *CYBB* gene	Electroporation	Base editing of autologous hematopoietic stem and progenitor cells (HSPCs) for ex vivo therapy. Designed to correct the c.676C>T mutation in the *CYBB* gene in HSPCs and later engraft them into patients. Modified HSPCs can differentiate into functional phagocytes with restored NADPH oxidase activity.
NCT06851767	Phase 1/2	Non-randomized, open-label, single-group assignment	18	Enrolling by invitation	X-linked severe combined immunodeficiency *IL2RG* gene	Electroporation	Base editing of autologous hematopoietic stem and progenitor cells (HSPCs) for ex vivo therapy. Designed to correct mutations in the *IL2RG* gene in different patients.
NCT06065189, NCT07000318, NCT06565026, NCT06024876, & NCT06479616	Phase 1	Open-label, single-arm	5	Active or recruiting	Major β—thalassemia and Severe sickle cell disease BCL11A binding site in Hemoglobin Subunit Gamma (HBG) promoter	Electroporation	Base editing of autologous hematopoietic stem cell transplantation (HSPCs) for ex vivo therapy. Designed to edit the HBG promoter in the BCL11A binding site to prevent the BCL11A inhibitory effect in γ-globin chain synthesis. This strategy is intended to increase fetal hemoglobin levels in the blood.
NCT06025032	Phase 1	Open-label, multiple-cohort, dose-finding	0	Withdrawn due to lack of patients in China	Auditory neuropathy (hearing loss) Nonsense mutation c.2485C>T (p. Q829X) in *OTOF* gene	AAV9 vector	In vivo AAV9-delivered CRISPR/Cas13 RNA base-editing therapy. Designed to correct c.2485C>T in *OTOF* gene and rescue otoferlin expression in sensory inner hair cells.
NCT07489196	Phase 2	Open-label, single-arm	20	Not yet recruiting	Major β—thalassemia BCL11A binding site in Hemoglobin Subunit Gamma (HBG) promoter	Electroporation	Base editing of autologous hematopoietic stem cell transplantation (HSPCs) for ex vivo therapy. Designed to edit the HBG promoter in the BCL11A binding site to prevent the BCL11A inhibitory effect in γ-globin chain synthesis. This strategy is intended to increase fetal hemoglobin levels in the blood.
NCT06594094	Phase 1	Open-label, multidose, dose-escalation	4	Completed	Duchenne muscular dystrophy Exon 51 splice donor site of *DMD* gene	AAV vector	In vivo AAV-delivered CRISPR/hfCas12Max base-editing therapy. Designed to edit the *DMD* exon 51 splice donor site to induce skipping of exon 51. Exon 51 skipping can restore the reading frame and recover dystrophin production in patients carrying different single- and multi-exon deletions.
NCT05398029	Phase 1	Open-label, single ascending-dose	13	Completed	Heterozygous familial hypercholesterolemia *PCSK9* gene (Splicing site)	Lipid nanoparticles	In vivo LNP-delivered CRISPR/ABE base-editing therapy. Designed to introduce a point mutation in the *PCSK9* splicing site to impair PCSK9 expression levels and reduce LDL-C levels in blood.
NCT06735755	Phase 1 & 2	Open-label, single-arm, ascending-dose, multicenter	36	Recruiting	Glycogen storage disease type-Ia (von Gierke disease) Missense mutation c.247C > T (p.R83C) in the *G6PC1* gene	Lipid nanoparticles	In vivo LNP- delivered CRISPR/ABE base-editing therapy. Designed to correct c.247C > T missense mutation in the *G6PC1* gene to rescue glucose-6-phosphatase-α production.
NCT05456880	Phase 1 & 2	Open-label, single-arm, multicenter	15	Recruiting	Sickle Cell Disease and Severe Vaso-Occlusive Crises *HBG1/2* gene promoters	Electroporation	Base editing of autologous CD34+ hematopoietic stem cells for ex vivo therapy. Designed to introduce mutations in the HBG1/2 gene promoters to disrupt BCL11A binding sites. This strategy is intended to increase fetal hemoglobin levels in the blood.
NCT06389877	Phase 1 & 2	Open-label, multicenter, dose-exploration and dose expansion	106	Recruiting	Alpha-1 antitrypsin deficiency (AATD) Missense mutation (E342K) in the *SERPINA1* gene	Lipid nanoparticles	In vivo LNP-delivered CRISPR/ABE base-editing therapy. Designed to correct the E342K point mutation in the *SERPINA1* gene to rescue α1-antitrypsin expression.
NCT06164730	Phase 1	Open-label, single-arm, ascending-dose	85	Recruiting	Familial Hypercholesterolemia or Premature Coronary Artery Disease *PCSK9* gene (Splicing site)	Lipid nanoparticles conjugated with GalNAc	In vivo LNP delivered CRISPR/ABE base-editing therapy. Designed to introduce a point mutation in the *PCSK9* splicing site to impair PCSK9 expression levels and reduce LDL-C levels in blood.
NCT06461702, & NCT06458010	Phase 1	Open-label, single-arm, single-dose escalation	13 & 20	Recruiting	Familial Hypercholesterolemia. Exon 1 splice donor site of *PCSK9.*	Lipid nanoparticles conjugated with GalNAc	In vivo LNP-delivered CRISPR/ hpABE5 base-editing therapy. Designed to introduce a point mutation in the *PCSK9* splicing site to impair PCSK9 expression levels and reduce LDL-C levels in blood.
NCT06451770	Phase 1	Open-label, single-arm, ascending-dose	36	Recruiting	Familial Hypercholesterolemia and Refractory Hyperlipidemia *ANGPTL3* gene	Lipid nanoparticles conjugated with GalNAc	In vivo LNP-delivered CRISPR/ABE8.8 base-editing therapy. Designed reduce expression of the *ANGPTL3* gene and reduce LDL-C and triglyceride levels in blood.
NCT06392724	Phase 1	Open-label, single-arm, single-center	3	Active, not recruiting	Duchenne muscular dystrophy 5′ splicing site of exon 50 of the *DMD* gene	Dual ss.AAV9 vector	In vivo AAV-delivered CRISPR/CBE base-editing therapy. Designed to edit the *DMD* exon 50 5′ splice site to induce skipping of exon 50. Exon 50 skipping can restore the reading frame and recover dystrophin production in patients carrying different single- and multi-exon deletions.
N/A	N/A	Single-patient, expanded-access Investigational New Drug application	1	N/A	Carbamoyl-phosphate synthetase 1 (CPS1) deficiency Missense mutation c.1003C→T (p.Gln335Ter) in *CPS1* gene	Lipid nanoparticles	In vivo personalized lipid nanoparticle-delivered CRISPR/ NGC-ABE8eV106W base-editing therapy. Designed to rescue carbamoyl-phosphate synthetase 1 production by correcting the c.1003C→T point mutation.

## Data Availability

No new data were created or analyzed in this study. All referenced data are available from the original publications, as listed in the references section.
